# Expression of c-erbB-2 protein in papillary thyroid carcinomas.

**DOI:** 10.1038/bjc.1992.177

**Published:** 1992-06

**Authors:** D. R. Haugen, L. A. Akslen, J. E. Varhaug, J. R. Lillehaug

**Affiliations:** Department of Biochemistry, University of Bergen, Norway.

## Abstract

**Images:**


 © Macmillan Press Ltd., 1992

### Expression of c-*erb*B-2 protein in papillary thyroid carcinomas

D.R.F. Haugen[1], L.A. Akslen[2], J.E. Varhaug[3] & J.R. Lillehaug[1]

[1]*Department of Biochemistry,* [2]*Department of Pathology and* [3]*Department of Surgery, University of Bergen, Bergen, Norway.*

**Summary** c-*erb*B-2 protein expression was investigated immunohistochemically in frozen thyroid tissue specimens from 42 patients using a polyclonal sheep antibody. c-*erb*B-2 immunoreactivity was detected in 12 out of 17 papillary carcinomas, while no c-*erb*B-2 protein immunostaining was seen in cases of follicular adenoma (five cases), follicular carcinoma (five cases) or medullary carcinoma (one case), nor in cases of non-neoplastic tissue, including normal thyroid tissue from tumour-bearing glands. RNA was extracted from 51 thyroid tissue samples from 34 of the above patients, and c-*erb*B-2 mRNA was analysed by slot-blot hybridisation. c-*erb*B-2 mRNA was detectable in all samples, but papillary carcinomas and lymph node metastases showed significantly higher levels of c-*erb*B-2 mRNA compared to non-neoplastic tissue.

The present demonstration of positive c-*erb*B-2 immunostaining in papillary thyroid carcinomas is contradictory to previous findings on formalin-fixed, paraffin-embedded material, and emphasises the importance of tissue quality for c-*erb*B-2 protein detection.

During recent years much has been done to elucidate the role of growth factors and oncogenes in the growth and function of normal thyroid follicular cells and in the development and maintenance of thyroid tumours.

The c-*erb*B-2 oncogene encodes a 185 kilodalton transmembrane glycoprotein with tyrosine kinase activity (Coussens *et al.*, 1985; Akiyama *et al.*, 1986). This protein is closely related to, but yet distinct from the EGF-receptor, encoded for by the c-*erb*B proto-oncogene (Schechter *et al.*, 1985). Recently, a ligand has been proposed for the putative c-*erb*B-2 growth factor receptor (Lupu *et al.*, 1990). The c-*erb*B-2 oncogene has been found to be amplified and/or overexpressed at mRNA or protein level in a number of human adenocarcinomas, those of the breast being most extensively studied (Slamon *et al.*, 1987; 1989; van de Vijver *et al.*, 1987; 1988; Venter *et al.*, 1987).

c-*erb*B-2 protein overexpression is currently being evaluated as a potential risk factor in breast cancer patients (Gullick *et al.*, 1991; Lovekin *et al.*, 1991; O'Reilly *et al.*, 1991; Winstanley *et al.*, 1991).

An analysis of c-*erb*B-2 and c-*erb*B mRNA expression in thyroid tumours by RNA slot blot hybridisation demonstrated two- to three-fold higher levels of c-*erb*B-2 and c-*erb*B RNA in three out of five papillary carcinomas and three papillary lymph node metastases, as compared to non-tumour tissue (Aasland *et al.*, 1988). The higher levels of expression of c-*erb*B and c-*erb*B-2 mRNA in the papillary carcinomas were much lower than the levels associated with gene amplification. Southern blot analysis showed no amplification or rearrangements of the c-*erb*B-2 gene (Aasland *et al.*, 1988). The results were pursued in a comprehensive analysis of the c-*erb*B and c-*erb*B-2 proto-oncogenes in human thyroid neoplasia, using Southern blot hybridisation to detect gene amplification or rearrangement, and immunocytochemistry to detect overexpression of c-*erb*B-2 oncoprotein (Lemoine *et al.*, 1990*a*). The Southern blot study showed no abnormality of either structure or gene copy number of the c-*erb*B or c-*erb*B-2 proto-oncogenes in 38 thyroid tumour samples, including 17 papillary carcinomas. Immunohistochemical staining of paraffin sections of 106 tumour specimens (24 papillary carcinomas) from pathological archives showed no cases of overexpression of c-*erb*B-2 proto-oncogene (Lemoine *et al.*, 1990*a*).

We have now extended these investigations by studying c-*erb*B-2 protein expression immunohistochemically in a series of thyroid tissue samples using fresh, frozen tissue, considering that a modest expression might be lost due to tissue processing when immunostaining is performed on formalin-fixed, paraffin-embedded material. In a number of the same tumours, c-*erb*B-2 mRNA expression has been analysed by slot blot hybridisation.

### Materials and methods

### *Tissue samples*

Fresh thyroid tissue was obtained from 42 patients subjected to either partial or total thyroidectomy between January 1990 and May 1991 at Haukeland University Hospital, Bergen, Norway. Immediately after excision, samples were cut from the surgical specimen(s), and each sample divided in two parts. One part was frozen directly in liquid nitrogen and stored at − 80°C for use in nucleic acid analysis.

The other part, intended for frozen sections, was immersed in Histocon transport medium (Histolab, Gothenburg, Sweden), and the following freezing procedure was completed within one hour. The pieces of tissue were embedded in Tissue Tek (Miles Scientific, Naperville, Il.) and frozen on cryostat bolts in isopentane precooled to liquid nitrogen temperature. The samples were stored at − 80°C.

The biopsies were classified according to conventional histopathological criteria, as defined by WHO (Hedinger, 1988), and the lesions included in this study, are listed in Table I. Two or more samples were obtained from each patient, the series comprising 115 samples from 42 patients in all. The histology of all specimens was examined by one of the authors (LAA).

### *Immunohistochemistry*

Sections were cut 6 μm thick in a frozen microtome, fixed in cold acetone for 10 min, and air dried. After a short rinse in PBS (0.01 M phosphate buffered 0.15 M saline, pH 7.3), sections were treated with 1% hydrogen peroxide ($H_2O_2$) in methanol for 30 min to block endogenous peroxidase activity, then rinsed in PBS again. After incubation for 30 min at room temperature with normal rabbit serum (Dakopatts, Copenhagen, Denmark) diluted 1:10 in PBS, sections were incubated overnight (18–22 h) at 4°C with a polyclonal sheep antibody to human c-*erb*B-2 oncoprotein (OA-11-854, batch no. 02846, Cambridge Research Biochemicals, Cambridge, UK). The antibody was used at dilution 1:500. After rinsing in PBS, sequential incubations with secondary antibody (biotinylated rabbit anti sheep IgG from Vector Laboratories, Burlingame, CA) at dilution 1:100 and AB-complex (10 μg $ml^{-1}$ avidin and 2.5 μg $ml^{-1}$ biotin-labelled peroxidase from Vectastain ABC kit, Vector

Correspondence: D.R.F. Haugen, Department of Biochemistry, University of Bergen, Årstadveien 19, N-5009 Bergen, Norway.
Received 19 November 1991; and in revised form 24 February 1992.

**Table I** Main histopathological diagnosis for patients included in c-*erb*B-2 protein immunohistochemical study

| *Histopathological diagnosis* | *No. of patients* |
|---|---|
| Diffuse hyperplasia (Thyreotoxicosis) | 5 |
| Colloid goitre | 11 |
| Follicular adenoma | 3 |
| Follicular carcinoma | 5 |
| Papillary carcinoma | 17[a] |
| Medullary carcinoma | 1 |

[a]Three patients presented only metastatic tissue.

Laboratories) followed, 30 min each, at room temperature. The sections were immersed in DAB colouring solution (0.03% 3′3 diaminobenzidine tetrahydrochloride [Sigma, St. Louis, Missouri] and 0.02% $H_2O_2$ in PBS) for 5 min, counterstained with haematoxylin, dehydrated and mounted. All dilutions of antibodies, normal serum and AB-complex were made with PBS containing 5% BSA (bovine serum albumin) as the diluent.

A negative control was included for each specimen, exchanging the primary antibody with PBS in duplicate sections (Figure 1f). Positive control specimens were used routinely to check the procedure. The immortalised human thyroid epithelial cell line SGHTL-34 (Whitley *et al.*,1987; Aasland *et al.*, 1990) was used as positive control. In our laboratory, this cell line has been shown to express c-*erb*B-2 (G.O. Ness and JRL, personal communication). Incubation of the primary antibody with the corresponding c-*erb*B-2 peptide (OP-11-3540, Cambridge Research Biochemicals) before applying on sections was done to ensure antibody specificity (Figure 1e). All controls gave satisfactory results.

To confirm the results, a second, monoclonal antibody to c-*erb*B-2 protein (OP15, lot no. 7900305, Oncogene Science, Manhasset, NY) was used. The staining procedure was performed as described above, except that the sections were incubated with the primary antibody at dilution 1:40 for 1 h, and the secondary antibody was biotinylated rabbit anti mouse IgG (Dakopatts) diluted 1:200.

**Figure 1** Immunohistochemical localisation of c-*erb*B-2 protein in frozen sections of papillary thyroid carcinomas, by the avidin-biotin-peroxidase method employing a polyclonal sheep antibody, as described in Materials and methods. **a**, Primary tumour with membrane reactivity; **b**, primary tumour with cytoplasmic reactivity; **c**, primary tumour without reactivity; **d**, lymph node metastasis with membrane and cytoplasmic reactivity; **e** and **f**, sections from the same tumour as in **d**, showing **e**, absence of staining following preincubation of antibody with peptide immunogen, and **f**, control in which primary antibody was omitted. Reduced by one third from × 560.

*RNA extraction*

RNA was extracted from 51 biopsies taken from 34 of the above patients, providing the following cases: six samples of histologically normal thyroid tissue (one sample taken from a patient with a follicular adenoma, the others taken from patients with papillary carcinomas), five diffuse hyperplasias, 12 colloid goitres, five follicular adenomas, four follicular carcinomas (three primary tumours and one metastasis), 11 papillary carcinomas (primary tumours) and eight lymph node metastases from papillary carcinomas.

Tissue samples were evacuated from liquid nitrogen, instantly minced and lysed in 4 M guanidinium isothiocyanate, as described by Aasland *et al.* (1988). Ultracentrifugation through a cesium chloride cushion was carried out at 27,000 r.p.m. for 18 h. Pelleted RNA was further processed as described (Aasland *et al.*, 1988).

*Slot blot analysis*

RNA was denatured at 56°C for 15 min in 20% formaldehyde and 30% 20 × SSC (standard saline-citrate) and applied to nylon membranes (NY 13N by Schleicher & Schuell, Dassel, Germany) in a vacuum slot blot apparatus (Schleicher & Schuell). For each case, a set of 2, 6 and 12 μg total RNA was applied, if there was a sufficient amount of RNA available. RNA concentrations were determined spectrophotometrically. As as internal control, some samples were included on all slot blot membranes. Prehybridisation and hybridisation were carried out in 50% formamide at 42°C in a hybridisation oven (Hybaid Ltd., Teddington, UK) as described (Sambrook *et al.*, 1989). DNA fragments were prepared from plasmids and $^{32}$P-labelled ([α-$^{32}$P]dCTP from Amersham, Aylesbury, UK) to high specific activity using the oligo-labelling technique (Feinberg & Vogelstein, 1983). The probe was a purified fragment of the cloned human c-*erb*B-2 gene, a partial c-*erb*B-2 cDNA 1.6 kbp EcoRI fragment of pCER 204 (Yamamoto *et al.*, 1986). Blots were washed to high stringency (65°C, 0.2 × SSC with 0.1% NaPPi and 0.1% SDS [sodium dodecyl sulphate]) and exposed on X-ray films (XAR 5 by Kodak, Rochester, NY) in the presence of intensifying screens at − 80°C.

After stripping of slot blot membranes in 0.1% SDS at 90°C for 7 min, hybridisation with a 28S rRNA probe was performed according to the same procedure. The 28S rRNA probe was the 1.4 kbp BamHI fragment of pA (Dr I.L. Gonzales, personal communication). Analysis of autoradiograms was performed by densitometric scanning using an Enhanced Laser Densitometer (LKB Products, Bromma, Sweden). The relative levels of c-*erb*B-2 mRNA expression were estimated from scanning results as the amount of radioactive probe hybridised to each RNA sample relative to the amount of 28S rRNA in each sample.

### Results

*Immunohistochemistry*

Employing the polyclonal sheep antibody OA-11-854, no c-*erb*B-2 protein immunostaining was seen in cases of colloid goitre, diffuse hyperplasia, follicular adenoma or follicular carcinoma, in the only medullary carcinoma included in the series nor in normal thyroid follicular epithelium, including microscopically normal tissue from tumour-bearing thyroid glands.

In the papillary carcinoma group, c-*erb*B-2 immunostaining was present in tumour samples from 12 of the 17 patients (Figure 1). Details on these patients are given in Table II. The positively stained samples included 11 out of 14 primary tumours. From three patients, tissue from the primary tumour was not available, but samples from lymph node metastases were obtained. In two of these cases, no immunoreactivity was found, while the third patient had immunopositive epithelium in all three nodes available.

**Table II** c-*erb*B-2 immunostaining in tissue samples from 17 patients with papillary thyroid carcinomas

| *Tissue category* | *No. of cases with positive c-*erb*B-2 immunostaining/ no. of cases analysed* |
|---|---|
| Primary tumour | 11/14 |
| Non-tumour tissue[a] | 0/12 |
| Follicular adenoma | 0/2 |
| Lymph node metastasis | 5/8 |

[a]Including normal and goitrous tissue.

Lymph node metastases were provided from five of the 14 primary tumours. Lymph nodes with positive c-*erb*B-2 immunostaining also had immunopositive primary tumours (four cases). In contrast, neither the primary tumour nor the metastasis showed c-*erb*B-2 immunoreactivity in one case.

Immunoreactivity was confined to tumour cells. The staining was specific and reproducible, although staining intensity was uniformly rather weak. In most cases, specific membrane staining as well as a weak cytoplasmic positivity of tumour cells were seen. Two cases showed a predominantly cytoplasmic reaction, while two cases demonstrated almost exclusively membrane staining. Two or more positive samples from the same patient (from different parts of the tumour or from metastases) showed the same staining pattern. The staining was homogeneously distributed in the sections. Apart from tumour cells, staining was seen in the colloid of thyroid follicles, independent of tissue diagnosis. Colloid staining was in most cases relatively strong.

Corresponding results were obtained using the monoclonal antibody OP15, with the exception that a weak cytoplasmic reaction was seen in one papillary carcinoma (patient no. 125) which did not show immunoreactivity when examined with the polyclonal antibody OA-11-854. With the monoclonal antibody, only cytoplasmic staining was present, while membrane staining as well as cytoplasmic reactivity were detectable with the polyclonal antibody.

*RNA analysis*

Relative amounts of c-*erb*B-2 mRNA from RNA slot blot hybridisation analysis are presented in Figure 2. An example of autoradiograms of slot blot membranes is given in Figure 3. The hybridisation experiments showed that papillary carcinomas and lymph node metastases expressed higher levels of c-*erb*B-2 mRNA relative to non-neoplastic tissue (normal thyroid tissue, diffuse hyperplasia and colloid goitre) (*t*-test: $P < 0.001$).

In two samples showing increased c-*erb*B-2 mRNA expression, the corresponding protein was not detected with the immunohistochemical assay (patients no. 91 and 114). The other cases showing increased c-*erb*B-2 mRNA expression also showed c-*erb*B-2 protein product expression immunohistochemically.

Three patients (no. 102, 117 and 122) showed c-*erb*B-2 protein immunoreactivity even though their c-*erb*B-2 mRNA levels were not different from those of the reference group (Figure 2).

In the papillary carcinoma group, two lymph node metastases (patients no. 136 and 139) demonstrated the lowest levels of c-*erb*B-2 mRNA. These were the only two papillary metastatic deposits included in the mRNA analysis in which no c-*erb*B-2 protein was detected.

### Discussion

In this study we provide evidence that c-*erb*B-2 protein expression is a feature of papillary thyroid carcinomas, in contrast to non-neoplastic thyroid tissue. We have investigated c-*erb*B-2 protein expression immunohistochemically in a series of thyroid tissue samples, using fresh, frozen tissue obtained directly during surgery, and a polyclonal antibody

**Figure 2** Relative levels of c-*erb*B-2 mRNA in thyroid tissue samples as measured by densitometric scanning of autoradiograms (see Materials and methods). The unit of expression is arbitrary. Tissue samples are denoted by the patient number followed by a sample number; the sample number is omitted when the value represents a mean of two equivalent samples. The biopsies are arranged by increasing level of expression, and are grouped according to histological classification as follows: FA: follicular adenoma; FC: follicular carcinoma; PC: papillary carcinoma; NT: normal thyroid tissue; CG: colloid goitre and DH: diffuse hyperplasia. * indicates lymph node metastasis, # indicates positive c-*erb*B-2 immunostaining.

**Figure 3** Example of autoradiograms from RNA slot blot hybridisation showing RNA expression of c-*erb*B-2 and 28S rRNA in tissue samples from papillary thyroid carcinomas (PC) and lymph node metastases from papillary carcinomas (LM). Samples of 6 $\mu$g RNA were slot blotted onto nylon membranes and hybridised to the probes indicated as described in Materials and methods.

to $p185^{c\text{-}erbB\text{-}2}$. Lemoine and coworkers were not able to detect any c-*erb*B-2 overexpression in 24 papillary carcinomas using formalin-fixed, paraffin-embedded material from pathological archives (Lemoine *et al.*, 1990*a*). Natali *et al.* (1990), however, demonstrated c-*erb*B-2 expression in two out of nine thyroid carcinomas (no further classification given) using frozen tissue. For breast cancer tissue, where c-*erb*B-2 expression has been most widely studied, the discrepancy between results from formalin-fixed and frozen material has been emphasised by Slamon *et al.* (1989), who reported that in virtually every case there was some reduction in immunohistochemical staining with polyclonal antiserum when comparing fixed to frozen tissue. In tumours expressing very high levels, the protein was visible by immunostaining in tissue prepared by either method. The problem was more significant in samples expressing moderate levels of protein since many of them completely lost their immunohistochemical reactivity during formalin fixation and paraffin embedding. Slamon concludes that the problem of loss of antigenic immunoreactivity during fixation can be overcome by using frozen tissue samples (Slamon *et al.*, 1989).

The difference between fixed and frozen material for c-*erb*B-2 oncoprotein detection has also been demonstrated for bladder cancer (Wright *et al.*, 1990). Recently, a novel monoclonal antibody to c-*erb*B-2 protein, NCL-CB11, has been introduced, and reported to be highly effective for immunohistochemistry using paraffin-embedded material. Even with this antibody, however, the authors cannot exclude that some immunopositive cases might be lost due to fixation (Corbett *et al.*, 1990).

Although several studies, using different antibodies, have demonstrated a significant correlation between c-*erb*B-2 gene amplification and immunohistochemical staining of c-*erb*B-2 protein (Venter *et al.*, 1987; Slamon *et al.*, 1989; Corbett *et al.*, 1990), evidence of overexpression has also been detected in breast tumours in which the gene copy number was determined to be single (Slamon *et al.*, 1989). In thyroid tumours, no c-*erb*B-2 gene amplification has been found (Aasland *et al.*, 1988; Lemoine *et al.*, 1990*a*), and it is therefore crucial to have optimal tissue quality and to exclude protein deteriorating procedures when looking for c-*erb*B-2 protein expression. In the present study, the staining intensity was uniformly rather weak, although specific and reproducible.

The majority of immunopositive cases showed a specific membrane staining as well as a weaker and more diffuse cytoplasmic reaction. Two cases demonstrated almost exclusively cytoplasmic staining, of stronger degree, and two cases exhibited membrane staining only. The membrane staining has been regarded as specific for c-*erb*B-2 protein expression in breast carcinomas, and this staining pattern is associated with gene amplification and of prognostic significance in these tumours (Gullick *et al.*, 1991; Lovekin *et al.*, 1991).

In other human tumours, however, different staining patterns have been observed. Diffuse cytoplasmic immunoreactivity was predominant in c-*erb*B-2 protein positive cases of pancreatic cancer (Hall *et al.*, 1990). In transitional cell carcinomas of the urinary bladder, cytoplasmic reactivity predominated, even in tumours with high levels of gene amplification (Coombs *et al.*, 1991). The significance of cytoplasmic staining has not yet been established. de Potter *et al.* (1989) showed that the cytoplasmic reacting protein was a protein of molecular weight 155 kD, different from the known $p185^{c\text{-}erbB\text{-}2}$. In bladder tumours with high c-*erb*B-2 gene copy number and mRNA expression and cytoplasmic staining, high levels of the 155 kD protein were detected (Coombs *et al.*, 1991). The close correlation of c-*erb*B-2 gene amplification and cytoplasmic immunoreactivity in transitional cell tumours argues that the cytoplasmic product does represent a form of the c-*erb*B-2 protein, possibly reflecting some alteration in processing or stability of the oncoprotein or its mRNA. In the present study, cytoplasmic as well as membrane staining was abolished when the primary antibody was preincubated with the immunising c-*erb*B-2 peptide.

Our series includes five follicular carcinomas. None of these stained positively in the immunohistochemical assay, but the number of cases is too small to draw any conclusions on c-*erb*B-2 expression in follicular thyroid carcinomas. The only medullary carcinoma included was also negative. Roncalli and coworkers found that none out of 28 medullary thyroid carcinomas displayed c-*erb*B-2 immunoreactivity using the monoclonal antibody N3, but the authors comment that fixation regimes might have adversely affected tumour immunoreactivity (Roncalli *et al.*, 1991).

c-*erb*B-2 mRNA was detected in all the samples analysed, and the levels of c-*erb*B-2 mRNA in papillary carcinomas and lymph node metastases were higher than the levels observed in non-neoplastic tissue, comprising the groups normal thyroid tissue, colloid goitre and diffuse hyperplasia. This observation is consistent with the previous findings by Aasland *et al.* (1988). The higher levels of c-*erb*B-2 mRNA in the papillary carcinoma group were much lower than the levels associated with gene amplification, in agreement with what would be expected from the findings by Lemoine *et al.* (1990*a*). It should, however, be kept in mind that our tumour samples contain variable amounts of non-neoplastic tissue. The contribution of c-*erb*B-2 mRNA and rRNA from non-neoplastic tissue to the total RNA isolated must therefore vary from specimen to specimen. Since rRNA most likely is increased in proliferating tumour cells compared to non-neoplastic cells, the increase in c-*erb*B-2 mRNA expression that has been found in the papillary carcinoma samples, may be underestimated. Consequently, the true increase in c-*erb*B-2 mRNA per tumour cell may be higher than we report.

The significant increase in c-*erb*B-2 mRNA expression in the papillary carcinoma group adds support to the protein expression data from the immunohistochemical analysis. The evidence of c-*erb*B-2 overexpression in the papillary carcinomas is, however, more clear from the immunohistochemical results than from the RNA slot blot hybridisation experiments. This is in agreement with Coombs and coworkers who reported that 40% of tumours with no detectable c-*erb*B-2 amplification or overexpression which could be detected by Northern or Western analysis, showed positive c-*erb*B-2 immunostaining (Coombs *et al.*, 1991). The conclusion from their work on bladder cancer is that immunocytochemistry may be the most sensitive assay for detection of c-*erb*B-2 expression. Slamon *et al.* (1989) also found that immunohistochemical analysis of c-*erb*B-2 protein in frozen tissue sections correlated best with all other analytic data for both breast and ovarian cancer.

The function of the c-*erb*B-2 protein in cell growth and development remains unknown. The protein has tyrosine kinase activity, and is postulated to be a transmembrane receptor (Yamamoto *et al.*, 1986), for which a ligand has not yet been fully established. The homology and close relationship to the EGF-receptor suggest that the c-*erb*B-2 protein may convey potent growth stimulatory signals. In human tumours, overexpression and not mutation of the c-*erb*B-2 gene seems to contribute to tumour development (Slamon *et al.*, 1989; Lemoine *et al.*, 1990*b*). In thyroid tumours also, no activating point mutations of the transmembrane-encoding region of the c-*erb*B-2 gene have been revealed (Lemoine *et al.*, 1990*a*).

Investigations on the role of growth factors and oncogenes in the development of thyroid tumours have revealed that activation of *ras* oncogenes (Lemoine *et al.*, 1989; Suarez *et al.*, 1990) and autocrine production of IGF-I (Williams *et al.*, 1988) occur in the early stages of thyroid follicular cell tumourigenesis, and TGF-$\beta$ expression is associated with the malignant stages (Jasani *et al.*, 1990). The nuclear oncogenes c-*myc* and c-*fos* have been found to be expressed at varying levels in both non-tumour and tumour tissue, but neither rearrangements nor amplifications of these oncogenes have been observed in several studies (Aasland *et al.*, 1988; Terrier *et al.*, 1988; Wyllie *et al.*, 1989). The PTC and *trk* tyrosine kinase oncogenes are activated in a number of papillary carcinomas (Fusco *et al.*, 1987; Bongarzone *et al.*, 1989), the PTC oncogene being a rearranged form of the *ret* oncogene (Grieco *et al.*, 1990).

The present work presents c-*erb*B-2 protein expression as a feature of papillary thyroid carcinomas, extending the list of human adenocarcinomas expressing this protein. The increased expression of c-*erb*B-2 protein in papillary thyroid carcinomas is not due to gene amplification, and no other genetic aberration explaining this increased expression has been identified. However, the large proportion of papillary thyroid carcinomas expressing the c-*erb*B-2 protein indicates a biologically significant mechanism involving this receptor system in papillary carcinomas. Further investigations will be needed to assign the biological significance and prognostic implications of c-*erb*B-2 protein expression in these thyroid tumours.

We thank Hilde Killingstad for excellent technical assistance, Gro Oddveig Ness for many helpful discussions and Ove Bruland for help in preparation of the diagram. This work was supported by the Norwegian Cancer Society, L. Meltzers høyskolefond and the Norwegian Research Council for Science and the Humanities.

### References

ASLAND, R., LILLEHAUG, J.R., MALE, R., JØSENDAL, O., VARHAUG, J.E. & KLEPPE, K. (1988). Expression of oncogenes in thyroid tumours: Coexpression of c-*erbB2/neu* and c-*erbB*. *Br. J. Cancer*, **57**, 358–363.

AASLAND, R., AKSLEN, L.A., VARHAUG, J.E. & LILLEHAUG, J.R. (1990). Co-expression of the genes encoding transforming growth factor-α and its receptor in papillary carcinomas of the thyroid. *Int. J. Cancer*, **46**, 382–387.

AKIYAMA, T., SUDO, C., OGAWARA, H., TOYOSHIMA, K. & YAMAMOTO, T. (1986). The Product of the Human c-*erb*B-2 Gene: A 185-Kilodalton Glycoprotein with Tyrosine Kinase Activity. *Science*, **232**, 1644–1646.

BONGARZONE, I., PIEROTTI, M.A., MONZINI, N., MONDELLINI, P., MANENTI, G., DONGHI, R., PILOTTI, S., GRIECO, M., SANTORO, M., FUSCO, A., VECCHIO, G. & DELLA PORTA, G. (1989). High frequency of activation of tyrosine kinase oncogenes in human papillary thyroid carcinoma. *Oncogene*, **4**, 1457–1462.

COOMBS, L.M., PIGOTT, D.A., SWEENEY, E., PROCTOR, A.J., EYDMANN, M.E., PARKINSON, C. & KNOWLES, M.A. (1991). Amplification and over-expression of c-*erb*B-2 in transitional cell carcinoma of the urinary bladder. *Br. J. Cancer*, **63**, 601–608.

CORBETT, I.P., HENRY, J.A., ANGUS, B., WATCHORN, C.J., WILKINSON, L., HENNESSY, C., GULLICK, W.J., TUZI, N.L., MAY, F.E.B., WESTLEY, B.R. & HORNE, C.H.W. (1990). NCL-CB11, a new monoclonal antibody recognizing the internal domain of the c-*erb*B-2 oncogene protein effective for use on formalin-fixed, paraffin-embedded tissue. *J. Pathol.*, **161**, 15–25.

COUSSENS, L., YANG-FENG, T.L., LIAO, Y.-C., CHEN, E., GRAY, A., MCGRATH, J., SEEBURG, P.H., LIBERMANN, T.A., SCHLESSINGER, J., FRANCKE, U., LEVINSON, A. & ULLRICH, A. (1985). Tyrosine kinase receptor with extensive homology to EGF receptor shares chromosomal location with *neu* oncogene. *Science*, **230**, 1132–1139.

DE POTTER, C.R., VAN DAELE, S., VAN DE VIJVER, M.J., PAUWELS, C., MAERTENS, G., DE BOEVER, J., VANDEKERCKHOVE, D. & ROELS, H. (1989). The expression of the *neu* oncogene product in breast lesions and in normal fetal and adult human tissues. *Histopathology*, **15**, 351–362.

FEINBERG, A.P. & VOGELSTEIN, B. (1983). A technique for radiolabeling DNA restriction endonuclease fragments to high specific activity. *Anal. Biochem.*, **132**, 6–13.

FUSCO, A., GRIECO, M., SANTORO, M., BERLINGIERI, M.T., PILOTTI, S., PIEROTTI, M.A., DELLA PORTA, G. & VECCHIO, G. (1987). A new oncogene in human thyroid papillary carcinomas and their lymph-nodal metastases. *Nature*, **328**, 170–172.

GRIECO, M., SANTORO, M., BERLINGIERI, M.T., MELILLO, R.M., DONGHI, R., BONGARZONE, I., PIEROTTI, M.A., DELLA PORTA, G., FUSCO, A. & VECCHIO, G. (1990). PTC is a novel rearranged form of the *ret* proto-oncogene and is frequently detected in vivo in human thyroid papillary carcinomas. *Cell*, **60**, 557–563.

GULLICK, W.J., LOVE, S.B., WRIGHT, C., BARNES, D.M., GUSTERSON, B., HARRIS, A.L. & ALTMAN, D.G. (1991). c-*erb*B-2 protein overexpression in breast cancer is a risk factor in patients with involved and uninvolved lymph nodes. *Br. J. Cancer*, **63**, 434–438.

HALL, P.A., HUGHES, C.M., STADDON, S.L., RICHMAN, P.I., GULLICK, W.J. & LEMOINE, N.R. (1990). The c-*erb*B-2 proto-oncogene in human pancreatic cancer. *J. Pathol.*, **161**, 195–200.

HEDINGER, C. (ed.) (1988). *Histological Typing of Thyroid Tumours*. 2nd edition. WHO. Springer-Verlag: Berlin.

JASANI, B., WYLLIE, F.S., WRIGHT, P.A., LEMOINE, N.R., WILLIAMS, E.D. & WYNFORD-THOMAS, D. (1990). Immunocytochemically detectable TGF-$\beta$ associated with malignancy in Thyroid epithelial neoplasia. *Growth Factors*, **2**, 149–155.

LEMOINE, N.R., MAYALL, E.S., WYLLIE, F.S. WILLIAMS, E.D., GOYNS, M., STRINGER, B. & WYNFORD-THOMAS, D. (1989). High frequency of *ras* oncogene activation in all stages of human thyroid tumorigenesis. *Oncogene*, **4**, 159–164.

LEMOINE, N.R., WYLLIE, F.S., LILLEHAUG, J.R., STADDON, S.L., HUGHES, C.M., AASLAND, R., SHAW, J., VARHAUG, J.E., BROWN, C.L., GULLICK, W.J. & WYNFORD-THOMAS, D. (1990*a*). Absence of abnormalities of the c-*erbB*-1 and c-*erbB*-2 proto-oncogenes in human thyroid neoplasia. *Eur. J. Cancer*, **26**, 777–779.

LEMOINE, N.R., STADDON, S., DICKSON, C., BARNES, D.M. & GULLICK, W.J. (1990*b*). Absence of activating transmembrane mutations in the c-*erbB-2* proto-oncogene in human breast cancer. *Oncogene*, **5**, 237–239.

LOVEKIN, C., ELLIS, I.O., LOCKER, A., ROBERTSON, J.F.R., BELL, J., NICHOLSON, R., GULLICK, W.J., ELSTON, C.W. & BLAMEY, R.W. (1991). c-*erb*B-2 oncoprotein expression in primary and advanced breast cancer. *Br. J. Cancer*, **63**, 439–443.

LUPU, R., COLOMER, R., ZUGMAIER, G., SARUP, J., SHEPARD, M., SLAMON, D. & LIPPMAN, M.E. (1990). Direct interaction of a ligand for the *erb*B2 oncogene product with the EGF receptor and $p185^{erbB2}$. *Science*, **249**, 1552–1555.

NATALI, P.G., NICOTRA, M.R., BIGOTTI, A., VENTURO, I., SLAMON, D.J., FENDLY, B.M. & ULLRICH, A. (1990). Expression of the p185 encoded by HER2 oncogene in normal and transformed human tissues. *Int. J. Cancer*, **45**, 457–461.

O'REILLY, S.M., BARNES, D.M., CAMPLEJOHN, R.S, BARTKOVA, J., GREGORY, W.M. & RICHARDS, M.A. (1991). The relationship between c-*erb*B-2 expression, S-phase fraction and prognosis in breast cancer. *Br. J. Cancer*, **63**, 444–446.

RONCALLI, M., SPRINGALL, D.R., VARNDELL, I.M., GAITONDE, V.V., HAMID, Q., IBRAHIM, N.B.N., GRIMELIUS, L., WILANDER, E., POLAK, J.M. & COGGI, G. (1991). Oncoprotein immunoreactivity in human endocrine tumours. *J. Pathol.*, **163**, 117–127.

SAMBROOK, J., FRITSCH, E.F. & MANIATIS, T. (1989). *Molecular Cloning*. Cold Spring Harbor Laboratory: New York.

SCHECHTER, A.L., HUNG, M.-C., VAIDYANATHAN, L., WEINBERG, R.A., YANG-FENG, T.L., FRANCKE, U., ULLRICH, A. & COUSSENS, L. (1985). The *neu* Gene: An *erb*B-Homologous Gene Distinct from and Unlinked to the Gene Encoding the EGF Receptor. *Science*, **229**, 976–978.

SLAMON, D.J., CLARK, G.M., WONG, S.G., LEVIN, W.J., ULLRICH, A. & MCGUIRE, W.L. (1987). Human breast cancer: correlation of relapse and survival with amplification of the HER-2/*neu* oncogene. *Science*, **235**, 177–182.

SLAMON, D.J., GODOLPHIN, W., JONES, L.A., HOLT, J.A., WONG, S.G., KEITH, D.E., LEVIN, W.J., STUART, S.G., UDOVE, J., ULLRICH, A. & PRESS, M.F. (1989). Studies of the HER-2/*neu* Proto-oncogene in human breast and ovarian cancer. *Science*, **244**, 707–712.

SUAREZ, H.G., DU VILLARD, J.A., CAILLOU, B., SCHLUMBERGER, M., TUBIANA, M., PARMENTIER, C. & MONIER, R. (1988). Detection of activated *ras* oncogenes in human thyroid carcinomas. *Oncogene*, **2**, 403–406.

TERRIER, P., SHENG, Z.-M., SCHLUMBERGER, M., TUBIANA, M., CAILLOUR, B., TRAVAGLI, J.-P., FRAGU, P., PARMENTIER, C. & RIOU, G. (1988). Structure and expression of c-*myc* and c-*fos* proto-oncogenes in thyroid carcinomas. *Br. J. Cancer*, **57**, 43–47.

VAN DE VIJVER, M., VAN DE BERSSLELAAR, R., DEVILEE, P., CORNELISSE, C., PETERSE, J. & NUSSE, R. (1987). Amplification of the *neu* (c-*erb*B-2) Oncogene in human mammary tumors is relatively frequent and is often accompanied by amplification of the linked c-*erbA* oncogene. *Mol. Cell. Biol.*, **7**, 2019–2023.

VAN DE VIJVER, M.J., MOOI, W.J., WISMAN, P., PETERSE, J.L. & NUSSE, R. (1988). Immunohistochemical detection of the *neu* protein in tissue sections of human breast tumors with amplified *neu* DNA. *Oncogene*, **2**, 175–178.

VENTER, D.J., TUZI, N.L., KUMAR, S. & GULLICK, W.J. (1987). Overexpression of the c-*erb*B-2 oncoprotein in human breast carcinomas: immunohistological assessment correlates with gene amplification. *Lancet*, **ii**, 69–72.

WHITLEY, G.SJ., NUSSEY, S.S. & JOHNSTONE, A.P. (1987). SGHTL-34, a thyrotrophin-responsive immortalised human thyroid cell line generated by transfection. *Mol. Cell. Endocrinol.*, **52**, 279–284.

WILLIAMS, D.W., WILLIAMS, E.D. & WYNFORD-THOMAS, D. (1988). Loss of dependence on IGF-1 for proliferation of human thyroid adenoma cells. *Br. J. Cancer*, **57**, 535–539.

WINSTANLEY, J., COOKE, T., MURRAY, G.D., PLATT-HIGGINS, A., GEORGE, W.D., HOLT, S., MYSKOV, M., SPEDDING, A., BARRACLOUGH, B.R. & RUDLAND, P.S. (1991). The long term prognostic significance of c-*erb*B-2 in primary breast cancer. *Br. J. Cancer*, **63**, 447–450.

WRIGHT, C., MELLON, K., NEAL, D.E., JOHNSTON, P., CORBETT, I.P. & HORNE, C.H.W. (1990). Expression of c-*erb*B-2 protein product in bladder cancer. *Br. J. Cancer*, **62**, 764–765.

WYLLIE, F.S., LEMOINE, N.R., WILLIAMS, E.D. & WYNFORD-THOMAS, D. (1989). Structure and expression of nuclear oncogenes in multi-stage thyroid tumori-genesis. *Br. J. Cancer*, **60**, 561–565.

YAMAMOTO, T., IKAWA, S., AKIYAMA, T., SEMBA, K., NOMURA, N., MIYAJIMA, N., SAITO, T. & TOYOSHIMA, K. (1986). Similarity of protein encoded by the human c-*erb-B*-2 gene to epidermal growth factor receptor. *Nature*, **319**, 230–234.


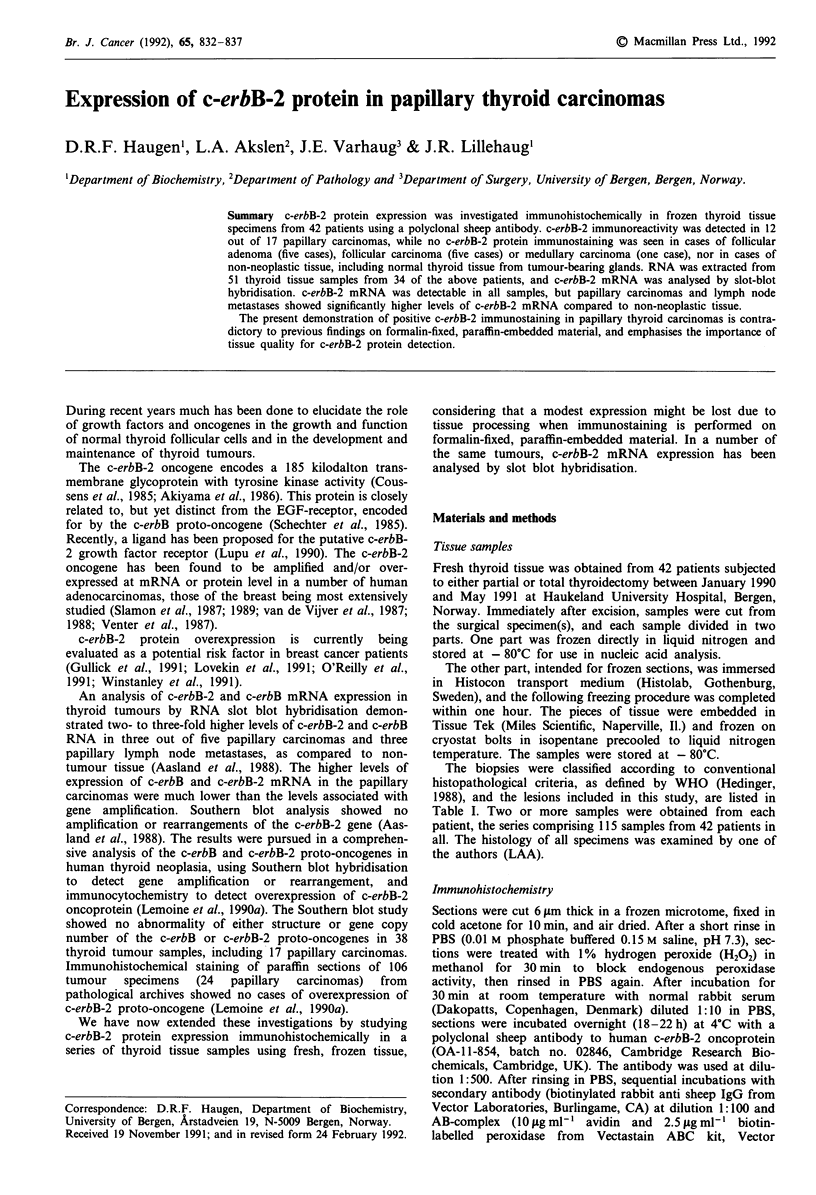

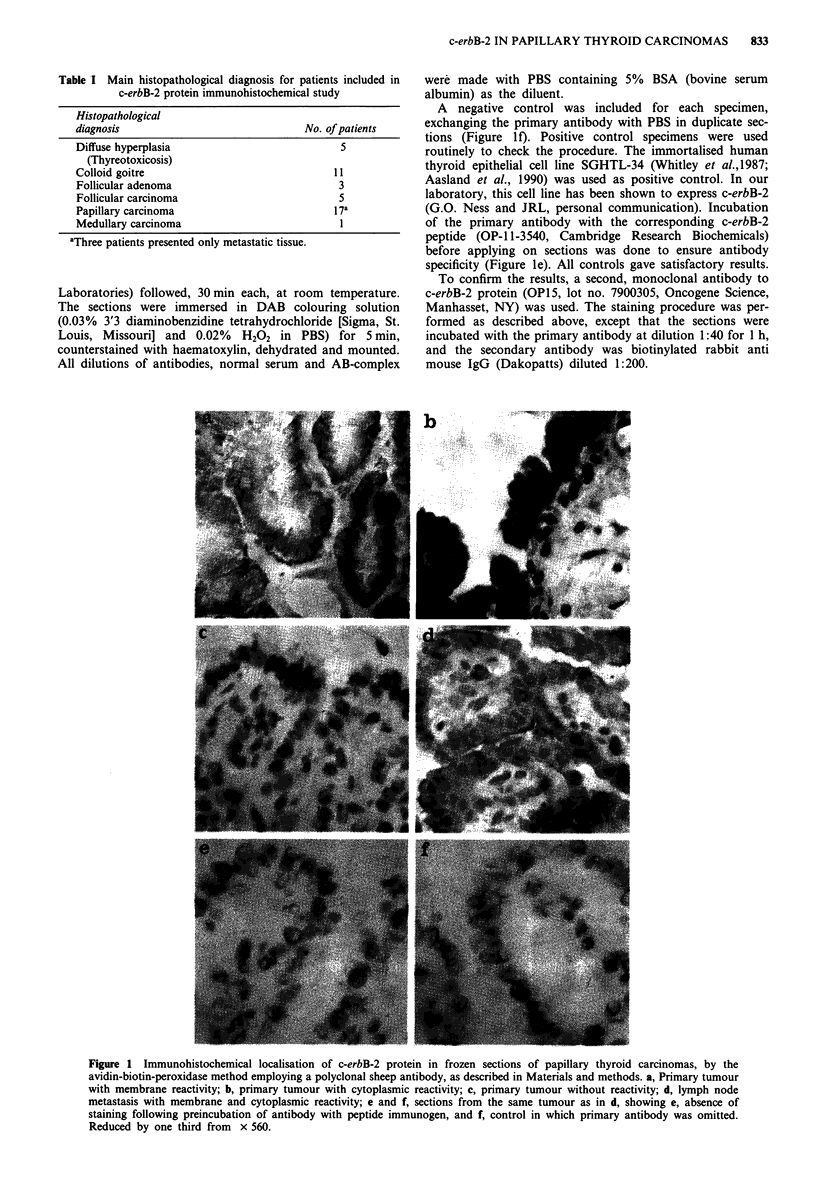

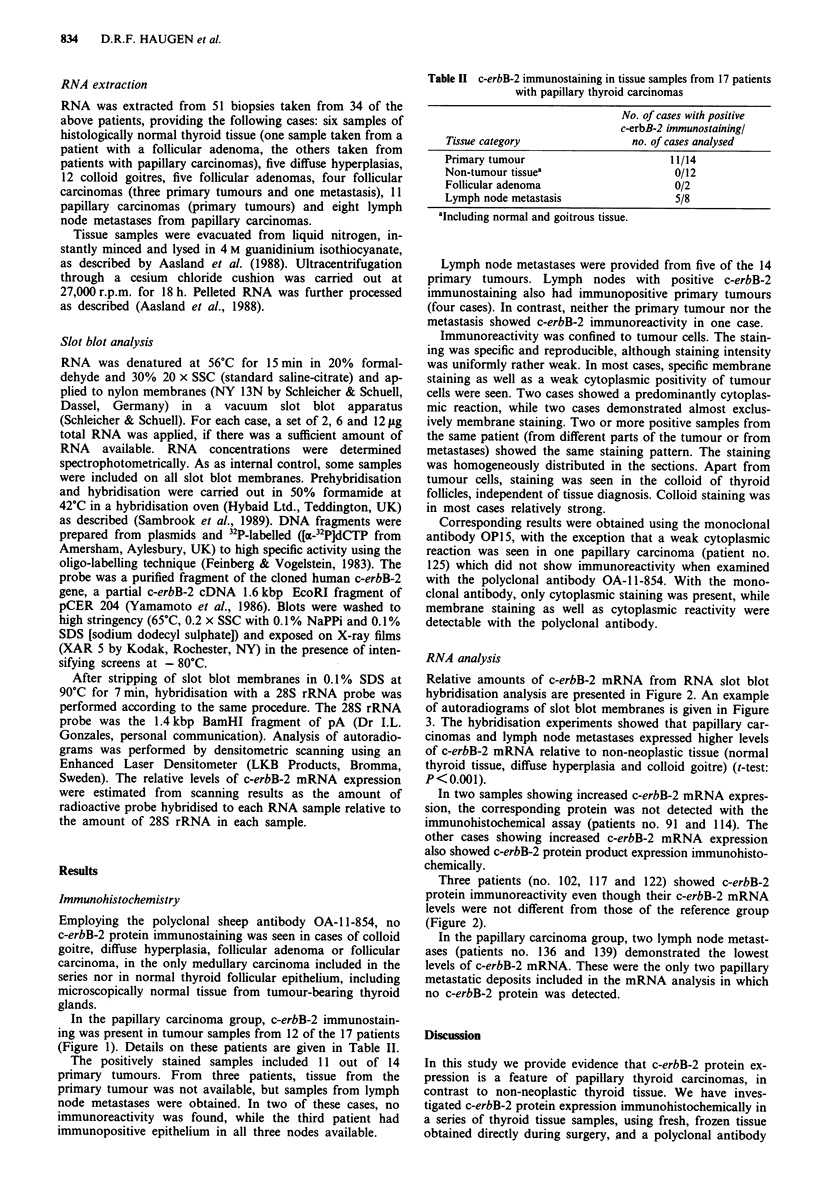

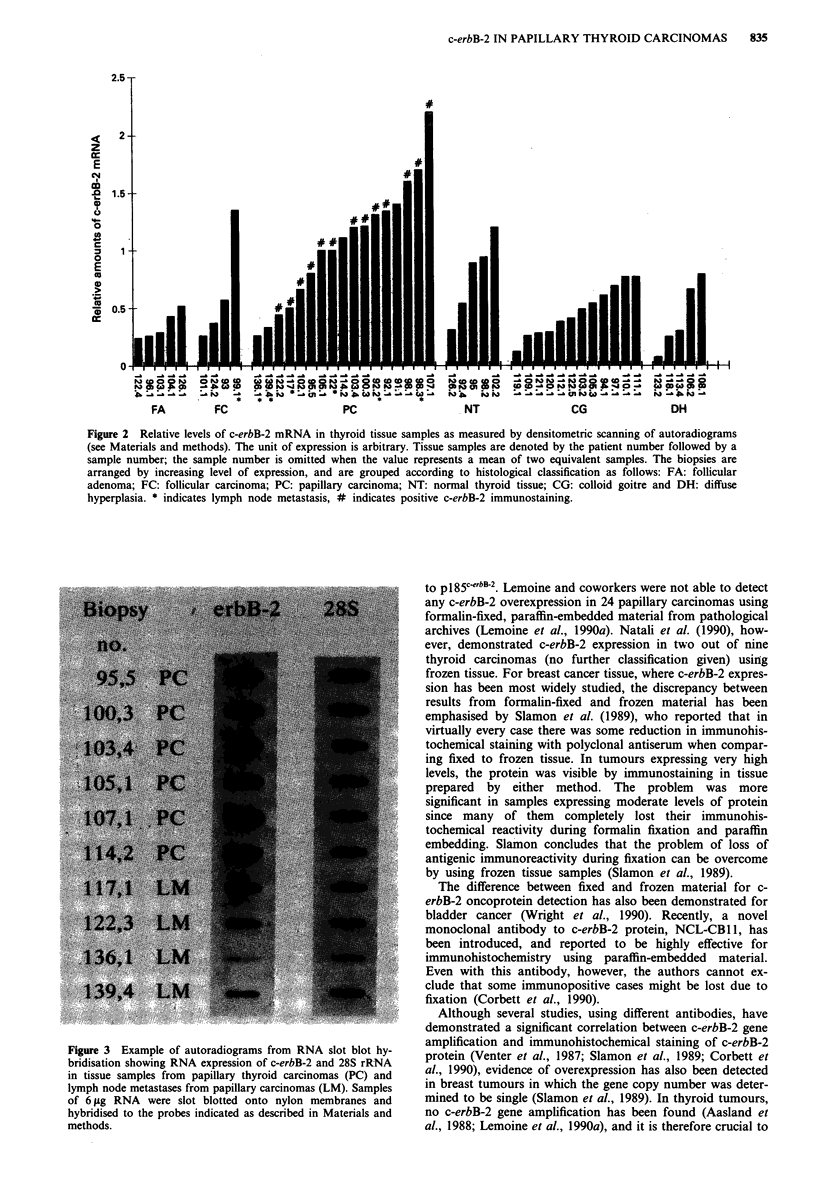

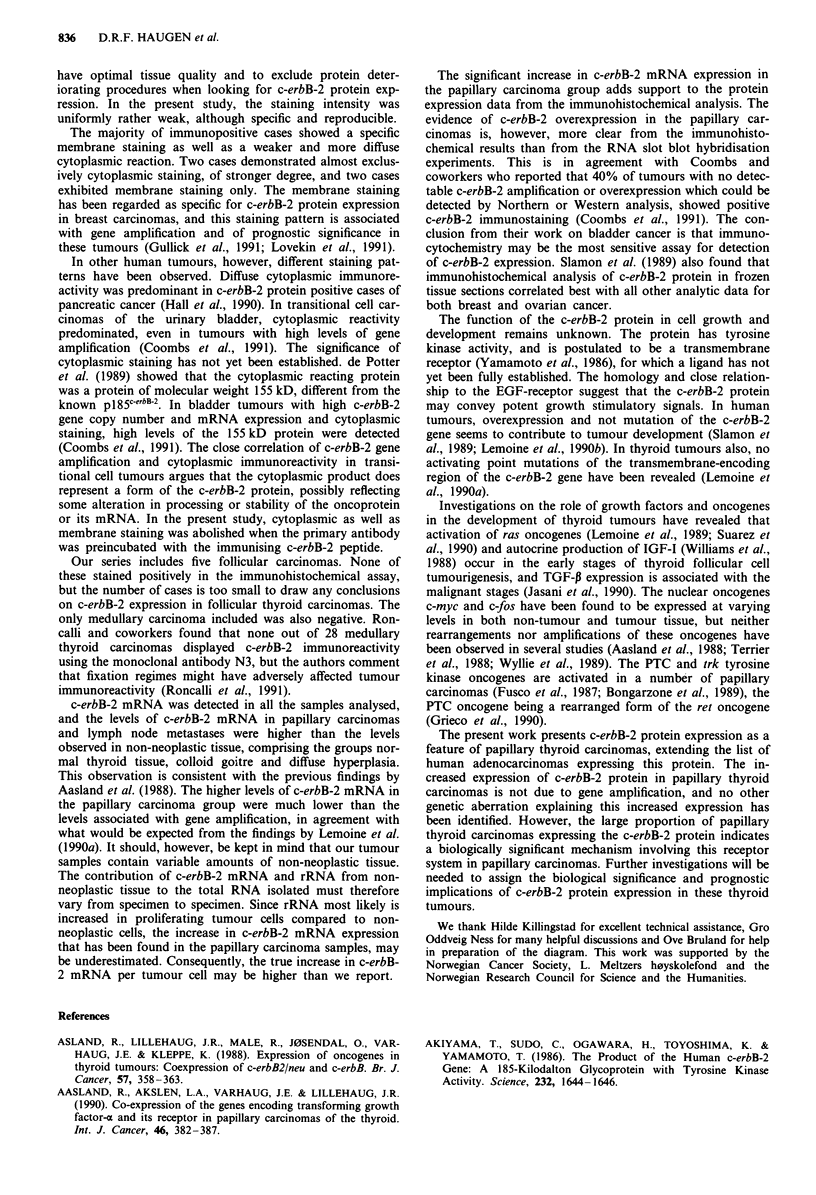

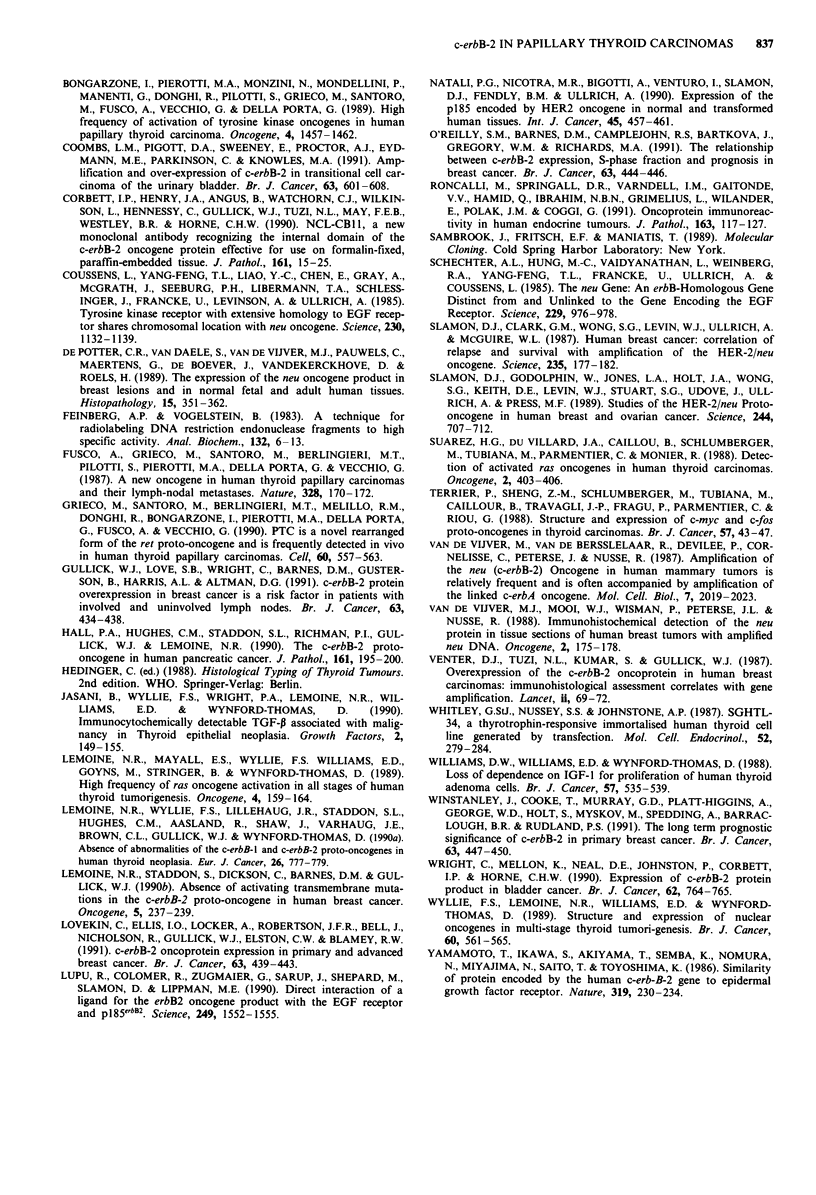


## References

[OCR_00612] Aasland R., Akslen L. A., Varhaug J. E., Lillehaug J. R. (1990). Co-expression of the genes encoding transforming growth factor-alpha and its receptor in papillary carcinomas of the thyroid.. Int J Cancer.

[OCR_00608] Aasland R., Lillehaug J. R., Male R., Jøsendal O., Varhaug J. E., Kleppe K. (1988). Expression of oncogenes in thyroid tumours: coexpression of c-erbB2/neu and c-erbB.. Br J Cancer.

[OCR_00618] Akiyama T., Sudo C., Ogawara H., Toyoshima K., Yamamoto T. (1986). The product of the human c-erbB-2 gene: a 185-kilodalton glycoprotein with tyrosine kinase activity.. Science.

[OCR_00626] Bongarzone I., Pierotti M. A., Monzini N., Mondellini P., Manenti G., Donghi R., Pilotti S., Grieco M., Santoro M., Fusco A. (1989). High frequency of activation of tyrosine kinase oncogenes in human papillary thyroid carcinoma.. Oncogene.

[OCR_00635] Coombs L. M., Pigott D. A., Sweeney E., Proctor A. J., Eydmann M. E., Parkinson C., Knowles M. A. (1991). Amplification and over-expression of c-erbB-2 in transitional cell carcinoma of the urinary bladder.. Br J Cancer.

[OCR_00641] Corbett I. P., Henry J. A., Angus B., Watchorn C. J., Wilkinson L., Hennessy C., Gullick W. J., Tuzi N. L., May F. E., Westley B. R. (1990). NCL-CB11, a new monoclonal antibody recognizing the internal domain of the c-erbB-2 oncogene protein effective for use on formalin-fixed, paraffin-embedded tissue.. J Pathol.

[OCR_00650] Coussens L., Yang-Feng T. L., Liao Y. C., Chen E., Gray A., McGrath J., Seeburg P. H., Libermann T. A., Schlessinger J., Francke U. (1985). Tyrosine kinase receptor with extensive homology to EGF receptor shares chromosomal location with neu oncogene.. Science.

[OCR_00655] De Potter C. R., Van Daele S., Van de Vijver M. J., Pauwels C., Maertens G., De Boever J., Vandekerckhove D., Roels H. (1989). The expression of the neu oncogene product in breast lesions and in normal fetal and adult human tissues.. Histopathology.

[OCR_00662] Feinberg A. P., Vogelstein B. (1983). A technique for radiolabeling DNA restriction endonuclease fragments to high specific activity.. Anal Biochem.

[OCR_00667] Fusco A., Grieco M., Santoro M., Berlingieri M. T., Pilotti S., Pierotti M. A., Della Porta G., Vecchio G. (1987). A new oncogene in human thyroid papillary carcinomas and their lymph-nodal metastases.. Nature.

[OCR_00673] Grieco M., Santoro M., Berlingieri M. T., Melillo R. M., Donghi R., Bongarzone I., Pierotti M. A., Della Porta G., Fusco A., Vecchio G. (1990). PTC is a novel rearranged form of the ret proto-oncogene and is frequently detected in vivo in human thyroid papillary carcinomas.. Cell.

[OCR_00682] Gullick W. J., Love S. B., Wright C., Barnes D. M., Gusterson B., Harris A. L., Altman D. G. (1991). c-erbB-2 protein overexpression in breast cancer is a risk factor in patients with involved and uninvolved lymph nodes.. Br J Cancer.

[OCR_00689] Hall P. A., Hughes C. M., Staddon S. L., Richman P. I., Gullick W. J., Lemoine N. R. (1990). The c-erb B-2 proto-oncogene in human pancreatic cancer.. J Pathol.

[OCR_00697] Jasani B., Wyllie F. S., Wright P. A., Lemoine N. R., Williams E. D., Wynford-Thomas D. (1990). Immunocytochemically detectable TGF-beta associated with malignancy in thyroid epithelial neoplasia.. Growth Factors.

[OCR_00704] Lemoine N. R., Mayall E. S., Wyllie F. S., Williams E. D., Goyns M., Stringer B., Wynford-Thomas D. (1989). High frequency of ras oncogene activation in all stages of human thyroid tumorigenesis.. Oncogene.

[OCR_00717] Lemoine N. R., Staddon S., Dickson C., Barnes D. M., Gullick W. J. (1990). Absence of activating transmembrane mutations in the c-erbB-2 proto-oncogene in human breast cancer.. Oncogene.

[OCR_00708] Lemoine N. R., Wyllie F. S., Lillehaug J. R., Staddon S. L., Hughes C. M., Aasland R., Shaw J., Varhaug J. E., Brown C. L., Gullick W. J. (1990). Absence of abnormalities of the c-erbB-1 and c-erbB-2 proto-oncogenes in human thyroid neoplasia.. Eur J Cancer.

[OCR_00721] Lovekin C., Ellis I. O., Locker A., Robertson J. F., Bell J., Nicholson R., Gullick W. J., Elston C. W., Blamey R. W. (1991). c-erbB-2 oncoprotein expression in primary and advanced breast cancer.. Br J Cancer.

[OCR_00727] Lupu R., Colomer R., Zugmaier G., Sarup J., Shepard M., Slamon D., Lippman M. E. (1990). Direct interaction of a ligand for the erbB2 oncogene product with the EGF receptor and p185erbB2.. Science.

[OCR_00733] Natali P. G., Nicotra M. R., Bigotti A., Venturo I., Slamon D. J., Fendly B. M., Ullrich A. (1990). Expression of the p185 encoded by HER2 oncogene in normal and transformed human tissues.. Int J Cancer.

[OCR_00739] O'Reilly S. M., Barnes D. M., Camplejohn R. S., Bartkova J., Gregory W. M., Richards M. A. (1991). The relationship between c-erbB-2 expression, S-phase fraction and prognosis in breast cancer.. Br J Cancer.

[OCR_00745] Roncalli M., Springall D. R., Varndell I. M., Gaitonde V. V., Hamid Q., Ibrahim N. B., Grimelius L., Wilander E., Polak J. M., Coggi G. (1991). Oncoprotein immunoreactivity in human endocrine tumours.. J Pathol.

[OCR_00755] Schechter A. L., Hung M. C., Vaidyanathan L., Weinberg R. A., Yang-Feng T. L., Francke U., Ullrich A., Coussens L. (1985). The neu gene: an erbB-homologous gene distinct from and unlinked to the gene encoding the EGF receptor.. Science.

[OCR_00762] Slamon D. J., Clark G. M., Wong S. G., Levin W. J., Ullrich A., McGuire W. L. (1987). Human breast cancer: correlation of relapse and survival with amplification of the HER-2/neu oncogene.. Science.

[OCR_00771] Slamon D. J., Godolphin W., Jones L. A., Holt J. A., Wong S. G., Keith D. E., Levin W. J., Stuart S. G., Udove J., Ullrich A. (1989). Studies of the HER-2/neu proto-oncogene in human breast and ovarian cancer.. Science.

[OCR_00775] Suárez H. G., Du Villard J. A., Caillou B., Schlumberger M., Tubiana M., Parmentier C., Monier R. (1988). Detection of activated ras oncogenes in human thyroid carcinomas.. Oncogene.

[OCR_00781] Terrier P., Sheng Z. M., Schlumberger M., Tubiana M., Caillou B., Travagli J. P., Fragu P., Parmentier C., Riou G. (1988). Structure and expression of c-myc and c-fos proto-oncogenes in thyroid carcinomas.. Br J Cancer.

[OCR_00799] Venter D. J., Tuzi N. L., Kumar S., Gullick W. J. (1987). Overexpression of the c-erbB-2 oncoprotein in human breast carcinomas: immunohistological assessment correlates with gene amplification.. Lancet.

[OCR_00805] Whitley G. S., Nussey S. S., Johnstone A. P. (1987). SGHTL-34, a thyrotrophin-responsive immortalised human thyroid cell line generated by transfection.. Mol Cell Endocrinol.

[OCR_00811] Williams D. W., Williams E. D., Wynford-Thomas D. (1988). Loss of dependence on IGF-1 for proliferation of human thyroid adenoma cells.. Br J Cancer.

[OCR_00819] Winstanley J., Cooke T., Murray G. D., Platt-Higgins A., George W. D., Holt S., Myskov M., Spedding A., Barraclough B. R., Rudland P. S. (1991). The long term prognostic significance of c-erbB-2 in primary breast cancer.. Br J Cancer.

[OCR_00823] Wright C., Mellon K., Neal D. E., Johnston P., Corbett I. P., Horne C. H. (1990). Expression of c-erbB-2 protein product in bladder cancer.. Br J Cancer.

[OCR_00830] Wyllie F. S., Lemoine N. R., Williams E. D., Wynford-Thomas D. (1989). Structure and expression of nuclear oncogenes in multi-stage thyroid tumorigenesis.. Br J Cancer.

[OCR_00834] Yamamoto T., Ikawa S., Akiyama T., Semba K., Nomura N., Miyajima N., Saito T., Toyoshima K. (1986). Similarity of protein encoded by the human c-erb-B-2 gene to epidermal growth factor receptor.. Nature.

[OCR_00793] van de Vijver M. J., Mooi W. J., Wisman P., Peterse J. L., Nusse R. (1988). Immunohistochemical detection of the neu protein in tissue sections of human breast tumors with amplified neu DNA.. Oncogene.

[OCR_00788] van de Vijver M., van de Bersselaar R., Devilee P., Cornelisse C., Peterse J., Nusse R. (1987). Amplification of the neu (c-erbB-2) oncogene in human mammmary tumors is relatively frequent and is often accompanied by amplification of the linked c-erbA oncogene.. Mol Cell Biol.

